# Supplementary material for: Pyruvate Dehydrogenase Kinase 1 inhibition mediated oxidative phosphorylation enhancement in cartilage promotes osteoarthritis progression
Source: BMC Musculoskelet Disord. 2023 Jul 20;24:597. doi: 10.1186/s12891-023-06585-6 (PMC10357736; doi:10.1186/s12891-023-06585-6)
Supplement: Supplementary file 1 — Additional file 1. [file 12891_2023_6585_MOESM1_ESM.zip › BP.Gsea.1653623667859/GOBP_NUCLEAR_TRANSPORT.html]

Details for gene set GOBP\_NUCLEAR\_TRANSPORT[GSEA]

|  || Dataset | 55457\_collapsed\_to\_symbols.cls.cls #OA\_versus\_normal.cls.cls #OA\_versus\_normal\_repos |
| Phenotype | cls.cls#OA\_versus\_normal\_repos |
| Upregulated in class | normal |
| GeneSet | GOBP\_NUCLEAR\_TRANSPORT |
| Enrichment Score (ES) | -0.34740537 |
| Normalized Enrichment Score (NES) | -1.7085075 |
| Nominal p-value | 0.0 |
| FDR q-value | 0.1297916 |
| FWER p-Value | 1.0 |
Table: GSEA Results Summary

  

Fig 1: Enrichment plot: GOBP\_NUCLEAR\_TRANSPORT      
 Profile of the Running ES Score & Positions of GeneSet Members on the Rank Ordered List

  

| SYMBOL | TITLE | RANK IN GENE LIST | RANK METRIC SCORE | RUNNING ES | CORE ENRICHMENT || 1 | ZIC1 | Zic family member 1 [Source:HGNC Symbol;Acc:HGNC:12872] | 0 | 1.459 | 0.0158 | No |
| 2 | PRKAG1 | protein kinase AMP-activated non-catalytic subunit gamma 1 [Source:HGNC Symbol;Acc:HGNC:9385] | 138 | 0.870 | 0.0145 | No |
| 3 | NMD3 | NMD3 ribosome export adaptor [Source:HGNC Symbol;Acc:HGNC:24250] | 240 | 0.791 | 0.0151 | No |
| 4 | HHEX | hematopoietically expressed homeobox [Source:HGNC Symbol;Acc:HGNC:4901] | 270 | 0.774 | 0.0212 | No |
| 5 | DRD1 | dopamine receptor D1 [Source:HGNC Symbol;Acc:HGNC:3020] | 392 | 0.709 | 0.0193 | No |
| 6 | ANP32A | acidic nuclear phosphoprotein 32 family member A [Source:HGNC Symbol;Acc:HGNC:13233] | 400 | 0.707 | 0.0264 | No |
| 7 | PSEN1 | presenilin 1 [Source:HGNC Symbol;Acc:HGNC:9508] | 425 | 0.695 | 0.0321 | No |
| 8 | XPO7 | exportin 7 [Source:HGNC Symbol;Acc:HGNC:14108] | 443 | 0.686 | 0.0382 | No |
| 9 | SEC13 | "SEC13 homolog, nuclear pore and COPII coat complex component [Source:HGNC Symbol;Acc:HGNC:10697]" | 460 | 0.677 | 0.0443 | No |
| 10 | SNUPN | snurportin 1 [Source:HGNC Symbol;Acc:HGNC:14245] | 477 | 0.670 | 0.0503 | No |
| 11 | APPL2 | "adaptor protein, phosphotyrosine interacting with PH domain and leucine zipper 2 [Source:HGNC Symbol;Acc:HGNC:18242]" | 586 | 0.628 | 0.0485 | No |
| 12 | NXT2 | nuclear transport factor 2 like export factor 2 [Source:HGNC Symbol;Acc:HGNC:18151] | 648 | 0.606 | 0.0503 | No |
| 13 | RANGRF | RAN guanine nucleotide release factor [Source:HGNC Symbol;Acc:HGNC:17679] | 660 | 0.603 | 0.0560 | No |
| 14 | PPP1CC | protein phosphatase 1 catalytic subunit gamma [Source:HGNC Symbol;Acc:HGNC:9283] | 744 | 0.583 | 0.0557 | No |
| 15 | RITA1 | RBPJ interacting and tubulin associated 1 [Source:HGNC Symbol;Acc:HGNC:25925] | 753 | 0.580 | 0.0614 | No |
| 16 | NEUROD1 | neuronal differentiation 1 [Source:HGNC Symbol;Acc:HGNC:7762] | 1008 | 0.515 | 0.0469 | No |
| 17 | AKT1 | AKT serine/threonine kinase 1 [Source:HGNC Symbol;Acc:HGNC:391] | 1192 | 0.478 | 0.0376 | No |
| 18 | RAB23 | "RAB23, member RAS oncogene family [Source:HGNC Symbol;Acc:HGNC:14263]" | 1225 | 0.471 | 0.0402 | No |
| 19 | HDAC3 | histone deacetylase 3 [Source:HGNC Symbol;Acc:HGNC:4854] | 1325 | 0.454 | 0.0373 | No |
| 20 | PCID2 | PCI domain containing 2 [Source:HGNC Symbol;Acc:HGNC:25653] | 1376 | 0.447 | 0.0382 | No |
| 21 | PARK7 | Parkinsonism associated deglycase [Source:HGNC Symbol;Acc:HGNC:16369] | 1406 | 0.442 | 0.0407 | No |
| 22 | HTATIP2 | HIV-1 Tat interactive protein 2 [Source:HGNC Symbol;Acc:HGNC:16637] | 1456 | 0.435 | 0.0416 | No |
| 23 | ANGPT1 | angiopoietin 1 [Source:HGNC Symbol;Acc:HGNC:484] | 1467 | 0.433 | 0.0455 | No |
| 24 | PKIA | cAMP-dependent protein kinase inhibitor alpha [Source:HGNC Symbol;Acc:HGNC:9017] | 1598 | 0.414 | 0.0397 | No |
| 25 | IFI27 | interferon alpha inducible protein 27 [Source:HGNC Symbol;Acc:HGNC:5397] | 1627 | 0.409 | 0.0419 | No |
| 26 | FGF9 | fibroblast growth factor 9 [Source:HGNC Symbol;Acc:HGNC:3687] | 1833 | 0.380 | 0.0298 | No |
| 27 | TRAF3IP2 | TRAF3 interacting protein 2 [Source:HGNC Symbol;Acc:HGNC:1343] | 1939 | 0.368 | 0.0255 | No |
| 28 | GAS6 | growth arrest specific 6 [Source:HGNC Symbol;Acc:HGNC:4168] | 1942 | 0.368 | 0.0293 | No |
| 29 | NUP133 | nucleoporin 133 [Source:HGNC Symbol;Acc:HGNC:18016] | 1951 | 0.367 | 0.0327 | No |
| 30 | RBM4 | RNA binding motif protein 4 [Source:HGNC Symbol;Acc:HGNC:9901] | 1970 | 0.364 | 0.0352 | No |
| 31 | PTTG1IP | PTTG1 interacting protein [Source:HGNC Symbol;Acc:HGNC:13524] | 2012 | 0.360 | 0.0359 | No |
| 32 | THOC7 | THO complex 7 [Source:HGNC Symbol;Acc:HGNC:29874] | 2170 | 0.341 | 0.0272 | No |
| 33 | PRKCD | protein kinase C delta [Source:HGNC Symbol;Acc:HGNC:9399] | 2237 | 0.335 | 0.0256 | No |
| 34 | MAPK14 | mitogen-activated protein kinase 14 [Source:HGNC Symbol;Acc:HGNC:6876] | 2352 | 0.320 | 0.0201 | No |
| 35 | RPAIN | RPA interacting protein [Source:HGNC Symbol;Acc:HGNC:28641] | 2450 | 0.308 | 0.0157 | No |
| 36 | XPO1 | exportin 1 [Source:HGNC Symbol;Acc:HGNC:12825] | 2477 | 0.305 | 0.0170 | No |
| 37 | TGFB1 | transforming growth factor beta 1 [Source:HGNC Symbol;Acc:HGNC:11766] | 2490 | 0.303 | 0.0193 | No |
| 38 | CDH1 | cadherin 1 [Source:HGNC Symbol;Acc:HGNC:1748] | 2589 | 0.292 | 0.0147 | No |
| 39 | POM121L2 | POM121 transmembrane nucleoporin like 2 [Source:HGNC Symbol;Acc:HGNC:13973] | 2841 | 0.266 | -0.0022 | No |
| 40 | GLI3 | GLI family zinc finger 3 [Source:HGNC Symbol;Acc:HGNC:4319] | 2861 | 0.264 | -0.0009 | No |
| 41 | ATXN1 | ataxin 1 [Source:HGNC Symbol;Acc:HGNC:10548] | 2862 | 0.264 | 0.0020 | No |
| 42 | HIKESHI | heat shock protein nuclear import factor hikeshi [Source:HGNC Symbol;Acc:HGNC:26938] | 2865 | 0.264 | 0.0047 | No |
| 43 | POLDIP3 | DNA polymerase delta interacting protein 3 [Source:HGNC Symbol;Acc:HGNC:23782] | 2869 | 0.264 | 0.0073 | No |
| 44 | BARD1 | BRCA1 associated RING domain 1 [Source:HGNC Symbol;Acc:HGNC:952] | 3051 | 0.245 | -0.0043 | No |
| 45 | RANBP6 | RAN binding protein 6 [Source:HGNC Symbol;Acc:HGNC:9851] | 3179 | 0.233 | -0.0118 | No |
| 46 | NUP93 | nucleoporin 93 [Source:HGNC Symbol;Acc:HGNC:28958] | 3194 | 0.231 | -0.0104 | No |
| 47 | STRADA | STE20 related adaptor alpha [Source:HGNC Symbol;Acc:HGNC:30172] | 3210 | 0.229 | -0.0091 | No |
| 48 | CDK5 | cyclin dependent kinase 5 [Source:HGNC Symbol;Acc:HGNC:1774] | 3400 | 0.211 | -0.0218 | No |
| 49 | EMD | emerin [Source:HGNC Symbol;Acc:HGNC:3331] | 3404 | 0.211 | -0.0197 | No |
| 50 | MDFIC | MyoD family inhibitor domain containing [Source:HGNC Symbol;Acc:HGNC:28870] | 3419 | 0.209 | -0.0186 | No |
| 51 | ANP32C | acidic nuclear phosphoprotein 32 family member C [Source:HGNC Symbol;Acc:HGNC:16675] | 3450 | 0.207 | -0.0187 | No |
| 52 | PKIG | cAMP-dependent protein kinase inhibitor gamma [Source:HGNC Symbol;Acc:HGNC:9019] | 3455 | 0.206 | -0.0168 | No |
| 53 | TRIM28 | tripartite motif containing 28 [Source:HGNC Symbol;Acc:HGNC:16384] | 3526 | 0.200 | -0.0202 | No |
| 54 | APPL1 | "adaptor protein, phosphotyrosine interacting with PH domain and leucine zipper 1 [Source:HGNC Symbol;Acc:HGNC:24035]" | 3532 | 0.199 | -0.0184 | No |
| 55 | ENY2 | ENY2 transcription and export complex 2 subunit [Source:HGNC Symbol;Acc:HGNC:24449] | 3561 | 0.197 | -0.0185 | No |
| 56 | RIOK2 | RIO kinase 2 [Source:HGNC Symbol;Acc:HGNC:18999] | 3615 | 0.192 | -0.0206 | No |
| 57 | FERMT1 | FERM domain containing kindlin 1 [Source:HGNC Symbol;Acc:HGNC:15889] | 3687 | 0.186 | -0.0242 | No |
| 58 | CDK1 | cyclin dependent kinase 1 [Source:HGNC Symbol;Acc:HGNC:1722] | 3744 | 0.182 | -0.0266 | No |
| 59 | KHDRBS1 | "KH RNA binding domain containing, signal transduction associated 1 [Source:HGNC Symbol;Acc:HGNC:18116]" | 3748 | 0.182 | -0.0249 | No |
| 60 | PPP3CA | protein phosphatase 3 catalytic subunit alpha [Source:HGNC Symbol;Acc:HGNC:9314] | 3803 | 0.176 | -0.0272 | No |
| 61 | NUP155 | nucleoporin 155 [Source:HGNC Symbol;Acc:HGNC:8063] | 3824 | 0.174 | -0.0269 | No |
| 62 | SMAD3 | SMAD family member 3 [Source:HGNC Symbol;Acc:HGNC:6769] | 4061 | 0.150 | -0.0440 | No |
| 63 | SYK | spleen associated tyrosine kinase [Source:HGNC Symbol;Acc:HGNC:11491] | 4084 | 0.147 | -0.0441 | No |
| 64 | PRKD1 | protein kinase D1 [Source:HGNC Symbol;Acc:HGNC:9407] | 4123 | 0.144 | -0.0456 | No |
| 65 | JAK2 | Janus kinase 2 [Source:HGNC Symbol;Acc:HGNC:6192] | 4183 | 0.140 | -0.0487 | No |
| 66 | SDAD1 | SDA1 domain containing 1 [Source:HGNC Symbol;Acc:HGNC:25537] | 4229 | 0.134 | -0.0508 | No |
| 67 | PHB2 | prohibitin 2 [Source:HGNC Symbol;Acc:HGNC:30306] | 4354 | 0.124 | -0.0593 | No |
| 68 | AGT | angiotensinogen [Source:HGNC Symbol;Acc:HGNC:333] | 4407 | 0.120 | -0.0621 | No |
| 69 | UPF1 | UPF1 RNA helicase and ATPase [Source:HGNC Symbol;Acc:HGNC:9962] | 4434 | 0.118 | -0.0629 | No |
| 70 | NUP42 | nucleoporin 42 [Source:HGNC Symbol;Acc:HGNC:17010] | 4479 | 0.115 | -0.0651 | No |
| 71 | DDX19B | DEAD-box helicase 19B [Source:HGNC Symbol;Acc:HGNC:2742] | 4500 | 0.113 | -0.0654 | No |
| 72 | NUP54 | nucleoporin 54 [Source:HGNC Symbol;Acc:HGNC:17359] | 4582 | 0.106 | -0.0707 | No |
| 73 | NUP85 | nucleoporin 85 [Source:HGNC Symbol;Acc:HGNC:8734] | 4630 | 0.101 | -0.0733 | No |
| 74 | RAE1 | ribonucleic acid export 1 [Source:HGNC Symbol;Acc:HGNC:9828] | 4697 | 0.096 | -0.0775 | No |
| 75 | TMCO6 | transmembrane and coiled-coil domains 6 [Source:HGNC Symbol;Acc:HGNC:28814] | 4727 | 0.093 | -0.0788 | No |
| 76 | MX2 | MX dynamin like GTPase 2 [Source:HGNC Symbol;Acc:HGNC:7533] | 4743 | 0.092 | -0.0790 | No |
| 77 | BACH2 | BTB domain and CNC homolog 2 [Source:HGNC Symbol;Acc:HGNC:14078] | 4993 | 0.074 | -0.0979 | No |
| 78 | NRDE2 | "NRDE-2, necessary for RNA interference, domain containing [Source:HGNC Symbol;Acc:HGNC:20186]" | 5019 | 0.071 | -0.0991 | No |
| 79 | TCF7L2 | transcription factor 7 like 2 [Source:HGNC Symbol;Acc:HGNC:11641] | 5034 | 0.070 | -0.0994 | No |
| 80 | ING1 | inhibitor of growth family member 1 [Source:HGNC Symbol;Acc:HGNC:6062] | 5146 | 0.060 | -0.1076 | No |
| 81 | EIF4ENIF1 | eukaryotic translation initiation factor 4E nuclear import factor 1 [Source:HGNC Symbol;Acc:HGNC:16687] | 5330 | 0.045 | -0.1215 | No |
| 82 | SUMO1 | small ubiquitin like modifier 1 [Source:HGNC Symbol;Acc:HGNC:12502] | 5337 | 0.045 | -0.1215 | No |
| 83 | TP53 | tumor protein p53 [Source:HGNC Symbol;Acc:HGNC:11998] | 5511 | 0.032 | -0.1349 | No |
| 84 | IPO13 | importin 13 [Source:HGNC Symbol;Acc:HGNC:16853] | 5640 | 0.021 | -0.1448 | No |
| 85 | NXF3 | nuclear RNA export factor 3 [Source:HGNC Symbol;Acc:HGNC:8073] | 5648 | 0.020 | -0.1451 | No |
| 86 | AHCYL1 | adenosylhomocysteinase like 1 [Source:HGNC Symbol;Acc:HGNC:344] | 5837 | 0.006 | -0.1599 | No |
| 87 | SP100 | SP100 nuclear antigen [Source:HGNC Symbol;Acc:HGNC:11206] | 5899 | 0.002 | -0.1647 | No |
| 88 | TPR | "translocated promoter region, nuclear basket protein [Source:HGNC Symbol;Acc:HGNC:12017]" | 5919 | 0.001 | -0.1662 | No |
| 89 | CCHCR1 | coiled-coil alpha-helical rod protein 1 [Source:HGNC Symbol;Acc:HGNC:13930] | 5954 | -0.002 | -0.1689 | No |
| 90 | NUP88 | nucleoporin 88 [Source:HGNC Symbol;Acc:HGNC:8067] | 5976 | -0.003 | -0.1705 | No |
| 91 | FRAT1 | FRAT regulator of WNT signaling pathway 1 [Source:HGNC Symbol;Acc:HGNC:3944] | 6027 | -0.007 | -0.1744 | No |
| 92 | GLE1 | GLE1 RNA export mediator [Source:HGNC Symbol;Acc:HGNC:4315] | 6058 | -0.009 | -0.1767 | No |
| 93 | EIF6 | eukaryotic translation initiation factor 6 [Source:HGNC Symbol;Acc:HGNC:6159] | 6140 | -0.015 | -0.1829 | No |
| 94 | SMG6 | SMG6 nonsense mediated mRNA decay factor [Source:HGNC Symbol;Acc:HGNC:17809] | 6175 | -0.019 | -0.1854 | No |
| 95 | SQSTM1 | sequestosome 1 [Source:HGNC Symbol;Acc:HGNC:11280] | 6260 | -0.025 | -0.1918 | No |
| 96 | IFNG | interferon gamma [Source:HGNC Symbol;Acc:HGNC:5438] | 6410 | -0.036 | -0.2032 | No |
| 97 | PML | PML nuclear body scaffold [Source:HGNC Symbol;Acc:HGNC:9113] | 6421 | -0.037 | -0.2036 | No |
| 98 | NUTF2 | nuclear transport factor 2 [Source:HGNC Symbol;Acc:HGNC:13722] | 6536 | -0.045 | -0.2121 | No |
| 99 | GCKR | glucokinase regulator [Source:HGNC Symbol;Acc:HGNC:4196] | 6576 | -0.048 | -0.2147 | No |
| 100 | WASHC4 | WASH complex subunit 4 [Source:HGNC Symbol;Acc:HGNC:29174] | 6612 | -0.050 | -0.2169 | No |
| 101 | NUP62 | nucleoporin 62 [Source:HGNC Symbol;Acc:HGNC:8066] | 6800 | -0.063 | -0.2310 | No |
| 102 | RBM15B | RNA binding motif protein 15B [Source:HGNC Symbol;Acc:HGNC:24303] | 6867 | -0.069 | -0.2355 | No |
| 103 | SETD2 | "SET domain containing 2, histone lysine methyltransferase [Source:HGNC Symbol;Acc:HGNC:18420]" | 6926 | -0.074 | -0.2393 | No |
| 104 | TSC2 | TSC complex subunit 2 [Source:HGNC Symbol;Acc:HGNC:12363] | 6946 | -0.075 | -0.2400 | No |
| 105 | IPO5 | importin 5 [Source:HGNC Symbol;Acc:HGNC:6402] | 6949 | -0.076 | -0.2393 | No |
| 106 | RANBP3 | RAN binding protein 3 [Source:HGNC Symbol;Acc:HGNC:9850] | 7043 | -0.083 | -0.2458 | No |
| 107 | AAAS | aladin WD repeat nucleoporin [Source:HGNC Symbol;Acc:HGNC:13666] | 7058 | -0.084 | -0.2459 | No |
| 108 | KPNA5 | karyopherin subunit alpha 5 [Source:HGNC Symbol;Acc:HGNC:6398] | 7062 | -0.085 | -0.2453 | No |
| 109 | NUP205 | nucleoporin 205 [Source:HGNC Symbol;Acc:HGNC:18658] | 7080 | -0.086 | -0.2457 | No |
| 110 | CHTOP | chromatin target of PRMT1 [Source:HGNC Symbol;Acc:HGNC:24511] | 7184 | -0.095 | -0.2528 | No |
| 111 | RANBP17 | RAN binding protein 17 [Source:HGNC Symbol;Acc:HGNC:14428] | 7194 | -0.096 | -0.2525 | No |
| 112 | UBR5 | ubiquitin protein ligase E3 component n-recognin 5 [Source:HGNC Symbol;Acc:HGNC:16806] | 7285 | -0.101 | -0.2585 | No |
| 113 | CALR | calreticulin [Source:HGNC Symbol;Acc:HGNC:1455] | 7291 | -0.102 | -0.2578 | No |
| 114 | NEDD4 | NEDD4 E3 ubiquitin protein ligase [Source:HGNC Symbol;Acc:HGNC:7727] | 7407 | -0.111 | -0.2657 | No |
| 115 | SIX2 | SIX homeobox 2 [Source:HGNC Symbol;Acc:HGNC:10888] | 7417 | -0.112 | -0.2652 | No |
| 116 | ELAVL1 | ELAV like RNA binding protein 1 [Source:HGNC Symbol;Acc:HGNC:3312] | 7426 | -0.113 | -0.2646 | No |
| 117 | FAM53C | family with sequence similarity 53 member C [Source:HGNC Symbol;Acc:HGNC:1336] | 7535 | -0.121 | -0.2718 | No |
| 118 | NUP62CL | nucleoporin 62 C-terminal like [Source:HGNC Symbol;Acc:HGNC:25960] | 7601 | -0.127 | -0.2756 | No |
| 119 | HCLS1 | hematopoietic cell-specific Lyn substrate 1 [Source:HGNC Symbol;Acc:HGNC:4844] | 7653 | -0.131 | -0.2782 | No |
| 120 | RGS14 | regulator of G protein signaling 14 [Source:HGNC Symbol;Acc:HGNC:9996] | 7719 | -0.135 | -0.2819 | No |
| 121 | POLR2D | RNA polymerase II subunit D [Source:HGNC Symbol;Acc:HGNC:9191] | 7726 | -0.136 | -0.2809 | No |
| 122 | NPAP1 | nuclear pore associated protein 1 [Source:HGNC Symbol;Acc:HGNC:1190] | 7737 | -0.136 | -0.2802 | No |
| 123 | CAMK1 | calcium/calmodulin dependent protein kinase I [Source:HGNC Symbol;Acc:HGNC:1459] | 7795 | -0.141 | -0.2831 | No |
| 124 | BMP4 | bone morphogenetic protein 4 [Source:HGNC Symbol;Acc:HGNC:1071] | 7816 | -0.142 | -0.2832 | No |
| 125 | TARDBP | TAR DNA binding protein [Source:HGNC Symbol;Acc:HGNC:11571] | 7864 | -0.146 | -0.2853 | No |
| 126 | CPSF6 | cleavage and polyadenylation specific factor 6 [Source:HGNC Symbol;Acc:HGNC:13871] | 7885 | -0.147 | -0.2853 | No |
| 127 | CD36 | CD36 molecule [Source:HGNC Symbol;Acc:HGNC:1663] | 7985 | -0.154 | -0.2915 | No |
| 128 | MCM3AP | minichromosome maintenance complex component 3 associated protein [Source:HGNC Symbol;Acc:HGNC:6946] | 8077 | -0.161 | -0.2969 | No |
| 129 | NOL6 | nucleolar protein 6 [Source:HGNC Symbol;Acc:HGNC:19910] | 8081 | -0.161 | -0.2954 | No |
| 130 | SFN | stratifin [Source:HGNC Symbol;Acc:HGNC:10773] | 8118 | -0.164 | -0.2965 | No |
| 131 | MALT1 | MALT1 paracaspase [Source:HGNC Symbol;Acc:HGNC:6819] | 8120 | -0.164 | -0.2948 | No |
| 132 | PPM1A | "protein phosphatase, Mg2+/Mn2+ dependent 1A [Source:HGNC Symbol;Acc:HGNC:9275]" | 8125 | -0.165 | -0.2933 | No |
| 133 | TXNIP | thioredoxin interacting protein [Source:HGNC Symbol;Acc:HGNC:16952] | 8254 | -0.175 | -0.3015 | No |
| 134 | KPNA1 | karyopherin subunit alpha 1 [Source:HGNC Symbol;Acc:HGNC:6394] | 8260 | -0.176 | -0.3000 | No |
| 135 | KPNB1 | karyopherin subunit beta 1 [Source:HGNC Symbol;Acc:HGNC:6400] | 8285 | -0.177 | -0.3000 | No |
| 136 | NUP214 | nucleoporin 214 [Source:HGNC Symbol;Acc:HGNC:8064] | 8301 | -0.178 | -0.2992 | No |
| 137 | FAM53B | family with sequence similarity 53 member B [Source:HGNC Symbol;Acc:HGNC:28968] | 8360 | -0.183 | -0.3018 | No |
| 138 | STAT3 | signal transducer and activator of transcription 3 [Source:HGNC Symbol;Acc:HGNC:11364] | 8453 | -0.190 | -0.3070 | No |
| 139 | NCBP2 | nuclear cap binding protein subunit 2 [Source:HGNC Symbol;Acc:HGNC:7659] | 8478 | -0.192 | -0.3068 | No |
| 140 | CRY2 | cryptochrome circadian regulator 2 [Source:HGNC Symbol;Acc:HGNC:2385] | 8536 | -0.196 | -0.3092 | No |
| 141 | THOC5 | THO complex 5 [Source:HGNC Symbol;Acc:HGNC:19074] | 8549 | -0.198 | -0.3080 | No |
| 142 | CHP2 | calcineurin like EF-hand protein 2 [Source:HGNC Symbol;Acc:HGNC:24927] | 8573 | -0.199 | -0.3077 | No |
| 143 | HNRNPA2B1 | heterogeneous nuclear ribonucleoprotein A2/B1 [Source:HGNC Symbol;Acc:HGNC:5033] | 8576 | -0.200 | -0.3057 | No |
| 144 | ATF2 | activating transcription factor 2 [Source:HGNC Symbol;Acc:HGNC:784] | 8592 | -0.201 | -0.3047 | No |
| 145 | ECT2 | epithelial cell transforming 2 [Source:HGNC Symbol;Acc:HGNC:3155] | 8647 | -0.204 | -0.3067 | No |
| 146 | SIRT7 | sirtuin 7 [Source:HGNC Symbol;Acc:HGNC:14935] | 8733 | -0.211 | -0.3112 | No |
| 147 | LEP | leptin [Source:HGNC Symbol;Acc:HGNC:6553] | 8740 | -0.212 | -0.3093 | No |
| 148 | SUPT6H | "SPT6 homolog, histone chaperone and transcription elongation factor [Source:HGNC Symbol;Acc:HGNC:11470]" | 8752 | -0.213 | -0.3079 | No |
| 149 | PKD1 | "polycystin 1, transient receptor potential channel interacting [Source:HGNC Symbol;Acc:HGNC:9008]" | 8755 | -0.213 | -0.3058 | No |
| 150 | DDX25 | DEAD-box helicase 25 [Source:HGNC Symbol;Acc:HGNC:18698] | 8815 | -0.218 | -0.3081 | No |
| 151 | SHH | sonic hedgehog signaling molecule [Source:HGNC Symbol;Acc:HGNC:10848] | 8941 | -0.227 | -0.3155 | No |
| 152 | UPF3B | UPF3B regulator of nonsense mediated mRNA decay [Source:HGNC Symbol;Acc:HGNC:20439] | 8986 | -0.230 | -0.3165 | No |
| 153 | CSE1L | chromosome segregation 1 like [Source:HGNC Symbol;Acc:HGNC:2431] | 9000 | -0.231 | -0.3150 | No |
| 154 | ZC3H12A | zinc finger CCCH-type containing 12A [Source:HGNC Symbol;Acc:HGNC:26259] | 9030 | -0.234 | -0.3147 | No |
| 155 | CABP1 | calcium binding protein 1 [Source:HGNC Symbol;Acc:HGNC:1384] | 9089 | -0.239 | -0.3167 | No |
| 156 | NCBP1 | nuclear cap binding protein subunit 1 [Source:HGNC Symbol;Acc:HGNC:7658] | 9149 | -0.244 | -0.3187 | No |
| 157 | ZPR1 | ZPR1 zinc finger [Source:HGNC Symbol;Acc:HGNC:13051] | 9151 | -0.244 | -0.3162 | No |
| 158 | HSPA9 | heat shock protein family A (Hsp70) member 9 [Source:HGNC Symbol;Acc:HGNC:5244] | 9173 | -0.246 | -0.3152 | No |
| 159 | NF1 | neurofibromin 1 [Source:HGNC Symbol;Acc:HGNC:7765] | 9194 | -0.247 | -0.3141 | No |
| 160 | KPNA6 | karyopherin subunit alpha 6 [Source:HGNC Symbol;Acc:HGNC:6399] | 9221 | -0.250 | -0.3134 | No |
| 161 | NUP107 | nucleoporin 107 [Source:HGNC Symbol;Acc:HGNC:29914] | 9274 | -0.253 | -0.3148 | No |
| 162 | MMP12 | matrix metallopeptidase 12 [Source:HGNC Symbol;Acc:HGNC:7158] | 9285 | -0.254 | -0.3128 | No |
| 163 | RAN | "RAN, member RAS oncogene family [Source:HGNC Symbol;Acc:HGNC:9846]" | 9478 | -0.272 | -0.3250 | No |
| 164 | TXN | thioredoxin [Source:HGNC Symbol;Acc:HGNC:12435] | 9487 | -0.273 | -0.3227 | No |
| 165 | NXF2 | nuclear RNA export factor 2 [Source:HGNC Symbol;Acc:HGNC:8072] | 9577 | -0.281 | -0.3267 | No |
| 166 | XPOT | exportin for tRNA [Source:HGNC Symbol;Acc:HGNC:12826] | 9597 | -0.283 | -0.3251 | No |
| 167 | ABCE1 | ATP binding cassette subfamily E member 1 [Source:HGNC Symbol;Acc:HGNC:69] | 9675 | -0.289 | -0.3281 | No |
| 168 | KPNA2 | karyopherin subunit alpha 2 [Source:HGNC Symbol;Acc:HGNC:6395] | 9753 | -0.297 | -0.3309 | No |
| 169 | XPO6 | exportin 6 [Source:HGNC Symbol;Acc:HGNC:19733] | 9759 | -0.297 | -0.3281 | No |
| 170 | FLNA | filamin A [Source:HGNC Symbol;Acc:HGNC:3754] | 9976 | -0.318 | -0.3417 | No |
| 171 | NXT1 | nuclear transport factor 2 like export factor 1 [Source:HGNC Symbol;Acc:HGNC:15913] | 9983 | -0.319 | -0.3387 | No |
| 172 | HSP90AB1 | heat shock protein 90 alpha family class B member 1 [Source:HGNC Symbol;Acc:HGNC:5258] | 10060 | -0.327 | -0.3412 | No |
| 173 | JUP | junction plakoglobin [Source:HGNC Symbol;Acc:HGNC:6207] | 10101 | -0.331 | -0.3408 | No |
| 174 | SMO | "smoothened, frizzled class receptor [Source:HGNC Symbol;Acc:HGNC:11119]" | 10111 | -0.332 | -0.3379 | No |
| 175 | SMG5 | SMG5 nonsense mediated mRNA decay factor [Source:HGNC Symbol;Acc:HGNC:24644] | 10190 | -0.340 | -0.3404 | No |
| 176 | SRSF3 | serine and arginine rich splicing factor 3 [Source:HGNC Symbol;Acc:HGNC:10785] | 10280 | -0.349 | -0.3436 | Yes |
| 177 | RANGAP1 | Ran GTPase activating protein 1 [Source:HGNC Symbol;Acc:HGNC:9854] | 10288 | -0.349 | -0.3404 | Yes |
| 178 | ANP32B | acidic nuclear phosphoprotein 32 family member B [Source:HGNC Symbol;Acc:HGNC:16677] | 10327 | -0.353 | -0.3395 | Yes |
| 179 | AXIN1 | axin 1 [Source:HGNC Symbol;Acc:HGNC:903] | 10335 | -0.354 | -0.3362 | Yes |
| 180 | RBM22 | RNA binding motif protein 22 [Source:HGNC Symbol;Acc:HGNC:25503] | 10345 | -0.355 | -0.3331 | Yes |
| 181 | KPNA3 | karyopherin subunit alpha 3 [Source:HGNC Symbol;Acc:HGNC:6396] | 10359 | -0.357 | -0.3303 | Yes |
| 182 | HYAL2 | hyaluronidase 2 [Source:HGNC Symbol;Acc:HGNC:5321] | 10420 | -0.363 | -0.3311 | Yes |
| 183 | UPF3A | UPF3A regulator of nonsense mediated mRNA decay [Source:HGNC Symbol;Acc:HGNC:20332] | 10455 | -0.368 | -0.3298 | Yes |
| 184 | RGPD5 | RANBP2 like and GRIP domain containing 5 [Source:HGNC Symbol;Acc:HGNC:32418] | 10604 | -0.384 | -0.3373 | Yes |
| 185 | PRKACA | protein kinase cAMP-activated catalytic subunit alpha [Source:HGNC Symbol;Acc:HGNC:9380] | 10611 | -0.384 | -0.3336 | Yes |
| 186 | APOD | apolipoprotein D [Source:HGNC Symbol;Acc:HGNC:612] | 10622 | -0.385 | -0.3302 | Yes |
| 187 | DHX9 | DExH-box helicase 9 [Source:HGNC Symbol;Acc:HGNC:2750] | 10627 | -0.386 | -0.3263 | Yes |
| 188 | STK3 | serine/threonine kinase 3 [Source:HGNC Symbol;Acc:HGNC:11406] | 10656 | -0.388 | -0.3243 | Yes |
| 189 | IPO7 | importin 7 [Source:HGNC Symbol;Acc:HGNC:9852] | 10662 | -0.389 | -0.3205 | Yes |
| 190 | YTHDC1 | YTH domain containing 1 [Source:HGNC Symbol;Acc:HGNC:30626] | 10687 | -0.391 | -0.3182 | Yes |
| 191 | UFM1 | ubiquitin fold modifier 1 [Source:HGNC Symbol;Acc:HGNC:20597] | 10783 | -0.404 | -0.3213 | Yes |
| 192 | NUP98 | nucleoporin 98 and 96 precursor [Source:HGNC Symbol;Acc:HGNC:8068] | 10875 | -0.413 | -0.3240 | Yes |
| 193 | SMG7 | SMG7 nonsense mediated mRNA decay factor [Source:HGNC Symbol;Acc:HGNC:16792] | 10877 | -0.414 | -0.3196 | Yes |
| 194 | XPO4 | exportin 4 [Source:HGNC Symbol;Acc:HGNC:17796] | 10912 | -0.419 | -0.3177 | Yes |
| 195 | CHP1 | calcineurin like EF-hand protein 1 [Source:HGNC Symbol;Acc:HGNC:17433] | 10940 | -0.423 | -0.3153 | Yes |
| 196 | NOLC1 | nucleolar and coiled-body phosphoprotein 1 [Source:HGNC Symbol;Acc:HGNC:15608] | 10942 | -0.423 | -0.3107 | Yes |
| 197 | LMNA | lamin A/C [Source:HGNC Symbol;Acc:HGNC:6636] | 10946 | -0.424 | -0.3064 | Yes |
| 198 | PIK3R2 | phosphoinositide-3-kinase regulatory subunit 2 [Source:HGNC Symbol;Acc:HGNC:8980] | 10956 | -0.425 | -0.3025 | Yes |
| 199 | FBXO22 | F-box protein 22 [Source:HGNC Symbol;Acc:HGNC:13593] | 11032 | -0.434 | -0.3037 | Yes |
| 200 | RRS1 | ribosome biogenesis regulator 1 homolog [Source:HGNC Symbol;Acc:HGNC:17083] | 11045 | -0.437 | -0.2999 | Yes |
| 201 | STK4 | serine/threonine kinase 4 [Source:HGNC Symbol;Acc:HGNC:11408] | 11074 | -0.441 | -0.2973 | Yes |
| 202 | PIK3R1 | phosphoinositide-3-kinase regulatory subunit 1 [Source:HGNC Symbol;Acc:HGNC:8979] | 11099 | -0.443 | -0.2944 | Yes |
| 203 | NUP160 | nucleoporin 160 [Source:HGNC Symbol;Acc:HGNC:18017] | 11100 | -0.444 | -0.2896 | Yes |
| 204 | IPO4 | importin 4 [Source:HGNC Symbol;Acc:HGNC:19426] | 11119 | -0.445 | -0.2862 | Yes |
| 205 | NUP58 | nucleoporin 58 [Source:HGNC Symbol;Acc:HGNC:20261] | 11132 | -0.447 | -0.2823 | Yes |
| 206 | MED1 | mediator complex subunit 1 [Source:HGNC Symbol;Acc:HGNC:9234] | 11146 | -0.450 | -0.2784 | Yes |
| 207 | GSK3B | glycogen synthase kinase 3 beta [Source:HGNC Symbol;Acc:HGNC:4617] | 11157 | -0.451 | -0.2743 | Yes |
| 208 | PPP1R12A | protein phosphatase 1 regulatory subunit 12A [Source:HGNC Symbol;Acc:HGNC:7618] | 11259 | -0.465 | -0.2772 | Yes |
| 209 | PTPN11 | protein tyrosine phosphatase non-receptor type 11 [Source:HGNC Symbol;Acc:HGNC:9644] | 11260 | -0.465 | -0.2722 | Yes |
| 210 | NUP153 | nucleoporin 153 [Source:HGNC Symbol;Acc:HGNC:8062] | 11307 | -0.472 | -0.2707 | Yes |
| 211 | DDX39A | DExD-box helicase 39A [Source:HGNC Symbol;Acc:HGNC:17821] | 11359 | -0.480 | -0.2695 | Yes |
| 212 | RAPGEF3 | Rap guanine nucleotide exchange factor 3 [Source:HGNC Symbol;Acc:HGNC:16629] | 11380 | -0.483 | -0.2659 | Yes |
| 213 | KPNA4 | karyopherin subunit alpha 4 [Source:HGNC Symbol;Acc:HGNC:6397] | 11395 | -0.486 | -0.2617 | Yes |
| 214 | BCL3 | BCL3 transcription coactivator [Source:HGNC Symbol;Acc:HGNC:998] | 11431 | -0.493 | -0.2591 | Yes |
| 215 | NCBP3 | nuclear cap binding subunit 3 [Source:HGNC Symbol;Acc:HGNC:24612] | 11502 | -0.501 | -0.2592 | Yes |
| 216 | BAG3 | BAG cochaperone 3 [Source:HGNC Symbol;Acc:HGNC:939] | 11562 | -0.509 | -0.2583 | Yes |
| 217 | TSC1 | TSC complex subunit 1 [Source:HGNC Symbol;Acc:HGNC:12362] | 11575 | -0.511 | -0.2537 | Yes |
| 218 | DESI1 | desumoylating isopeptidase 1 [Source:HGNC Symbol;Acc:HGNC:24577] | 11617 | -0.519 | -0.2513 | Yes |
| 219 | RPS15 | ribosomal protein S15 [Source:HGNC Symbol;Acc:HGNC:10388] | 11671 | -0.527 | -0.2498 | Yes |
| 220 | ZC3H11A | zinc finger CCCH-type containing 11A [Source:HGNC Symbol;Acc:HGNC:29093] | 11780 | -0.546 | -0.2524 | Yes |
| 221 | LSG1 | large 60S subunit nuclear export GTPase 1 [Source:HGNC Symbol;Acc:HGNC:25652] | 11783 | -0.546 | -0.2466 | Yes |
| 222 | PTPN14 | protein tyrosine phosphatase non-receptor type 14 [Source:HGNC Symbol;Acc:HGNC:9647] | 11797 | -0.548 | -0.2417 | Yes |
| 223 | DDX19A | DEAD-box helicase 19A [Source:HGNC Symbol;Acc:HGNC:25628] | 11821 | -0.553 | -0.2375 | Yes |
| 224 | NUP188 | nucleoporin 188 [Source:HGNC Symbol;Acc:HGNC:17859] | 11877 | -0.567 | -0.2357 | Yes |
| 225 | HEATR3 | HEAT repeat containing 3 [Source:HGNC Symbol;Acc:HGNC:26087] | 11901 | -0.572 | -0.2313 | Yes |
| 226 | THOC6 | THO complex 6 [Source:HGNC Symbol;Acc:HGNC:28369] | 11918 | -0.576 | -0.2263 | Yes |
| 227 | SSB | small RNA binding exonuclease protection factor La [Source:HGNC Symbol;Acc:HGNC:11316] | 11955 | -0.584 | -0.2228 | Yes |
| 228 | TNPO3 | transportin 3 [Source:HGNC Symbol;Acc:HGNC:17103] | 12032 | -0.600 | -0.2223 | Yes |
| 229 | AGFG1 | ArfGAP with FG repeats 1 [Source:HGNC Symbol;Acc:HGNC:5175] | 12035 | -0.602 | -0.2160 | Yes |
| 230 | IL1B | interleukin 1 beta [Source:HGNC Symbol;Acc:HGNC:5992] | 12105 | -0.622 | -0.2147 | Yes |
| 231 | NUP50 | nucleoporin 50 [Source:HGNC Symbol;Acc:HGNC:8065] | 12127 | -0.626 | -0.2095 | Yes |
| 232 | YWHAE | tyrosine 3-monooxygenase/tryptophan 5-monooxygenase activation protein epsilon [Source:HGNC Symbol;Acc:HGNC:12851] | 12137 | -0.628 | -0.2034 | Yes |
| 233 | POM121 | POM121 transmembrane nucleoporin [Source:HGNC Symbol;Acc:HGNC:19702] | 12146 | -0.631 | -0.1972 | Yes |
| 234 | UPF2 | UPF2 regulator of nonsense mediated mRNA decay [Source:HGNC Symbol;Acc:HGNC:17854] | 12150 | -0.631 | -0.1906 | Yes |
| 235 | RSRC1 | arginine and serine rich coiled-coil 1 [Source:HGNC Symbol;Acc:HGNC:24152] | 12165 | -0.635 | -0.1848 | Yes |
| 236 | TNPO2 | transportin 2 [Source:HGNC Symbol;Acc:HGNC:19998] | 12227 | -0.652 | -0.1825 | Yes |
| 237 | SMG1 | SMG1 nonsense mediated mRNA decay associated PI3K related kinase [Source:HGNC Symbol;Acc:HGNC:30045] | 12268 | -0.668 | -0.1784 | Yes |
| 238 | SMURF1 | SMAD specific E3 ubiquitin protein ligase 1 [Source:HGNC Symbol;Acc:HGNC:16807] | 12344 | -0.688 | -0.1769 | Yes |
| 239 | HNRNPA1 | heterogeneous nuclear ribonucleoprotein A1 [Source:HGNC Symbol;Acc:HGNC:5031] | 12356 | -0.693 | -0.1702 | Yes |
| 240 | NXF1 | nuclear RNA export factor 1 [Source:HGNC Symbol;Acc:HGNC:8071] | 12410 | -0.710 | -0.1667 | Yes |
| 241 | PABPN1 | poly(A) binding protein nuclear 1 [Source:HGNC Symbol;Acc:HGNC:8565] | 12461 | -0.730 | -0.1628 | Yes |
| 242 | CITED1 | Cbp/p300 interacting transactivator with Glu/Asp rich carboxy-terminal domain 1 [Source:HGNC Symbol;Acc:HGNC:1986] | 12493 | -0.746 | -0.1571 | Yes |
| 243 | THOC1 | THO complex 1 [Source:HGNC Symbol;Acc:HGNC:19070] | 12503 | -0.749 | -0.1497 | Yes |
| 244 | NPM1 | nucleophosmin 1 [Source:HGNC Symbol;Acc:HGNC:7910] | 12530 | -0.762 | -0.1435 | Yes |
| 245 | NEMF | nuclear export mediator factor [Source:HGNC Symbol;Acc:HGNC:10663] | 12531 | -0.762 | -0.1352 | Yes |
| 246 | NOTCH1 | notch receptor 1 [Source:HGNC Symbol;Acc:HGNC:7881] | 12555 | -0.776 | -0.1286 | Yes |
| 247 | RPL23 | ribosomal protein L23 [Source:HGNC Symbol;Acc:HGNC:10316] | 12558 | -0.777 | -0.1203 | Yes |
| 248 | PPP1R10 | protein phosphatase 1 regulatory subunit 10 [Source:HGNC Symbol;Acc:HGNC:9284] | 12568 | -0.780 | -0.1125 | Yes |
| 249 | AKAP13 | A-kinase anchoring protein 13 [Source:HGNC Symbol;Acc:HGNC:371] | 12572 | -0.782 | -0.1043 | Yes |
| 250 | IPO9 | importin 9 [Source:HGNC Symbol;Acc:HGNC:19425] | 12575 | -0.783 | -0.0959 | Yes |
| 251 | RANBP2 | RAN binding protein 2 [Source:HGNC Symbol;Acc:HGNC:9848] | 12609 | -0.804 | -0.0898 | Yes |
| 252 | PTGS2 | prostaglandin-endoperoxide synthase 2 [Source:HGNC Symbol;Acc:HGNC:9605] | 12641 | -0.820 | -0.0834 | Yes |
| 253 | MAVS | mitochondrial antiviral signaling protein [Source:HGNC Symbol;Acc:HGNC:29233] | 12646 | -0.822 | -0.0747 | Yes |
| 254 | AKAP8L | A-kinase anchoring protein 8 like [Source:HGNC Symbol;Acc:HGNC:29857] | 12665 | -0.836 | -0.0671 | Yes |
| 255 | THOC2 | THO complex 2 [Source:HGNC Symbol;Acc:HGNC:19073] | 12691 | -0.855 | -0.0598 | Yes |
| 256 | CBLB | Cbl proto-oncogene B [Source:HGNC Symbol;Acc:HGNC:1542] | 12725 | -0.887 | -0.0528 | Yes |
| 257 | CDKN1A | cyclin dependent kinase inhibitor 1A [Source:HGNC Symbol;Acc:HGNC:1784] | 12727 | -0.890 | -0.0432 | Yes |
| 258 | IPO8 | importin 8 [Source:HGNC Symbol;Acc:HGNC:9853] | 12761 | -0.924 | -0.0357 | Yes |
| 259 | DDX39B | DExD-box helicase 39B [Source:HGNC Symbol;Acc:HGNC:13917] | 12783 | -0.941 | -0.0272 | Yes |
| 260 | NFKBIA | NFKB inhibitor alpha [Source:HGNC Symbol;Acc:HGNC:7797] | 12817 | -1.001 | -0.0189 | Yes |
| 261 | TNPO1 | transportin 1 [Source:HGNC Symbol;Acc:HGNC:6401] | 12864 | -1.108 | -0.0105 | Yes |
| 262 | E2F3 | E2F transcription factor 3 [Source:HGNC Symbol;Acc:HGNC:3115] | 12886 | -1.224 | 0.0011 | Yes |
Table: GSEA details [plain text format]

  

Fig 2: GOBP\_NUCLEAR\_TRANSPORT      
 Blue-Pink O' Gram in the Space of the Analyzed GeneSet

  

Fig 3: GOBP\_NUCLEAR\_TRANSPORT: Random ES distribution      
 Gene set null distribution of ES for **GOBP\_NUCLEAR\_TRANSPORT**
